# The impact of endoxifen-guided tamoxifen dose reductions on endocrine side-effects in patients with primary breast cancer

**DOI:** 10.1016/j.esmoop.2023.100786

**Published:** 2023-02-06

**Authors:** S.M. Buijs, E. Oomen-de Hoop, C.L. Braal, M.M. van Rosmalen, J.C. Drooger, Q.C. van Rossum-Schornagel, M.B. Vastbinder, S.L.W. Koolen, A. Jager, R.H.J. Mathijssen

**Affiliations:** 1 Department of Medical Oncology, Erasmus MC Cancer Institute, Rotterdam, The Netherlands; 2 Department of Medical Oncology, Breast Cancer Center South Holland South, Ikazia Hospital, Rotterdam, The Netherlands; 3 Department of Internal Medicine, Franciscus Gasthuis & Vlietland, Schiedam, The Netherlands; 4 Department of Internal Medicine, IJsselland Hospital, Capelle aan den Ijssel, Rotterdam, The Netherlands; 5 Department of Hospital Pharmacy, Erasmus University Medical Center, Rotterdam, The Netherlands

**Keywords:** early breast cancer, endoxifen, side-effects, therapeutic drug monitoring, dose reduction

## Abstract

**Background:**

Tamoxifen is important in the adjuvant treatment of hormone-sensitive breast cancer and substantially reduces recurrence; however, almost 50% of patients are non-compliant mainly due to side-effects. The aim of this study was to investigate whether endoxifen-guided tamoxifen dose reduction could lead to fewer side-effects.

**Materials and methods:**

Effects of tamoxifen dose reduction were investigated in patients with bothersome side-effects and endoxifen levels ≥32 nM and compared to patients with side-effects who remained on tamoxifen 20 mg. Endocrine symptoms and health-related quality of life (HR-QOL) were assessed after 3 months with the Functional Assessment of Cancer Therapy—Endocrine Symptoms (FACT-ES) questionnaire.

**Results:**

Tamoxifen dose was reduced in 20 patients, 17 of whom were assessable for side-effect analyses. A clinically relevant improvement of *>*6 points was observed in endocrine symptoms and HR-QOL in 41% and 65% of the patients, respectively. In total, there was a significant and clinically relevant improvement in endocrine symptoms [5.7, 95% confidence interval (CI) −0.5-11.5] and HR-QOL (8.2, 95% CI 0.9-15.4) after dose reduction. This was not seen in patients whose doses were not reduced (*n* = 60). In 21% of patients, endoxifen dropped slightly below the 16-nM threshold (12.8, 15.5, 15.8, 15.9 nM).

**Conclusions:**

Endoxifen-guided dose reduction of tamoxifen significantly improved tamoxifen-related side-effects and HR-QOL. Nearly 80% of patients remained above the most conservative endoxifen threshold.

## Highlights

• Almost half of the patients with primary breast cancer discontinue tamoxifen early, mainly due to side-effects.
• Endoxifen-guided dose reduction in case of tamoxifen-related side-effects can improve symptoms in almost half of patients.
• Also, endoxifen-guided dose reduction strongly increased HR-QOL in two-thirds of patients.
• These improvements were not seen over time in patients with side-effects who remained on tamoxifen 20 mg.
• Endoxifen remained above or around the conservative threshold of 16 nM in the majority of dose-reduced patients.

## Introduction

Tamoxifen is currently recommended in the adjuvant treatment of early estrogen receptor-positive breast cancer for 5-10 years for premenopausal women and for 2-3 years for postmenopausal women.^1^ Unfortunately, during this long treatment period many patients experience tamoxifen-related side-effects such as hot flashes, arthralgia, vaginal dryness, mood alterations and insomnia.^2^ Therefore, almost 20% of all patients stop tamoxifen treatment already in the first year of therapy and another annual 5%-10% of patients are non-compliant for the remainder of the treatment period.^3^^,^^4^ For patients remaining on tamoxifen therapy, a substantial proportion of them have side-effects impacting their quality of life.^5^ Despite the high incidence of this problem, there is currently a lack of successful interventions in case patients experience tamoxifen-related side-effects.

A possible solution could be to carry out dose reductions in patients who experience many side-effects. From earlier research—where tamoxifen was prescribed to patients with high mammographic density—we already know that the incidence of tamoxifen-related side-effects was ≈50% lower with tamoxifen 1-5 mg daily compared to tamoxifen 20 mg daily.^6^ In another study with patients with ductal carcinoma *in situ*, tamoxifen 5 mg daily led to side-effects which were equal to placebo.^7^ Also, a study with tamoxifen in the preventive setting showed that the tamoxifen discontinuation rate of tamoxifen 5 mg daily was almost two-thirds lower than when tamoxifen 20 mg was prescribed.^8^ Thus, in the non-invasive setting, lower dosing of tamoxifen seems effective in reducing side-effects. However, surprisingly, in primary breast cancer patients on adjuvant tamoxifen, the effect of dose reduction on side-effects has hardly been studied.

Tamoxifen is a prodrug and is metabolized in, mainly, endoxifen, the metabolite that contributes for the most part to the antiestrogenic effect of tamoxifen.^9^^,^^10^ Endoxifen can be measured easily in plasma.^11^ There are several retrospective studies suggesting an exposure–response relationship for endoxifen.^12^, ^13^, ^14^ An analysis including 1370 patients receiving adjuvant tamoxifen found that patients in the lowest endoxifen exposure quintile (up to 16 nM) had a 26% higher risk of recurrence than patients in the other four endoxifen exposure quintiles.^12^ When exploring dichotomized cut-off points for a lower risk of recurrence, again an endoxifen threshold of 16 nM was found.^12^ Two other small studies found a higher risk of recurrence in patients with endoxifen levels below 14 nM and below 9 nM, respectively.^13^^,^^14^ The threshold of 16 nM (i.e. 5.97 ng/ml) is most generally accepted in the field of precision dosing of tamoxifen, since it has been found in the largest study thus far and is the most conservative threshold, at which the likelihood of patients being underdosed is assumed negligible.^12^^,^^15^^,^^16^ This endoxifen threshold could be used to carry out a responsible tamoxifen dose reduction in patients who experience bothersome tamoxifen-related side-effects. Only one study investigated halving the tamoxifen dose in the adjuvant setting before, but this study only focussed on severe hot flashes and was not guided by endoxifen levels.^17^

Therefore, the aim of the present study was to investigate whether endoxifen-guided dose reduction of tamoxifen in patients with bothersome tamoxifen-related side-effects could lead to fewer side-effects and better quality of life while retaining adequate endoxifen levels.

## Materials and methods

The TOTAM (Therapeutic drug monitoring Of TAMoxifen) trial is a large intervention study coordinated by the Erasmus MC Cancer Institute in Rotterdam, the Netherlands. This study was approved by the local Medical Ethics Committee in January 2018 and registered in the International Clinical Trial Registry Platform (ICTRP; https://trialsearch.who.int/; NL6918). Informed consent was obtained from all participants. The main goal of the TOTAM study was to investigate the feasibility of therapeutic drug monitoring of tamoxifen. A secondary endpoint of this trial was to investigate the effect of reducing the tamoxifen dose in patients with bothersome side-effects and a steady-state endoxifen plasma level of 32 nM or higher (i.e. two times the threshold of 16 nM).

### Patients and study design

Female patients who were using tamoxifen in the standard daily dose of 20 mg for 3 months were included in the TOTAM study. The design of the study and the main results have been described in detail elsewhere.^18^^,^^19^ For the current research question, all patients had to fill in the Functional Assessment of Cancer Therapy—Endocrine Symptoms (FACT-ES) questionnaire at baseline (=3 months of tamoxifen) and after 6 weeks (=4.5 months of tamoxifen) and 3 months (=6 months of tamoxifen). During the study, a dose reduction of tamoxifen from 20 mg to 10 mg was proposed to patients who experienced bothersome subjective side-effects which impacted their quality of life or wherefore they were considering discontinuation of tamoxifen. Simultaneously, they had to have an endoxifen level ≥32 nM. Also, the endocrine symptoms (ES19) score of the FACT-ES questionnaire had to be ≤72 points (maximum: 76 points) in order to be able to measure a clinically relevant difference in ES19 (i.e. 4 points) after dose reduction. Patients were seen at 3 months, 4.5 months and 6 months of tamoxifen use or, when these time points did not coincide, at 6 weeks and 3 months after tamoxifen dose reduction.

### Pharmacokinetic analysis

Tamoxifen and endoxifen trough levels (C_min_ concentrations) were obtained during every study visit. Plasma levels were measured using a validated ultra-performance liquid chromatography with a tandem mass spectrometry method (UP-LCMS/MS).^11^

### Quality of life and side-effect analysis

Also, during every study visit, the toxicity of tamoxifen was assessed using the US National Cancer Institute’s Common Terminology Criteria for Adverse Events version 5 (CTCAEv5) and quality of life and tamoxifen-related side-effects were evaluated using the FACT-ES questionnaire. The FACT-ES is a validated and reliable questionnaire of in total 46 questions and is a measure of health-related quality of life (HR-QOL, 27 items) using physical, social, emotional and functional well-being questions and a measure of side-effects of endocrine treatments given in breast cancer patients (ES19, endocrine subscale of in total 19 items). Four other endocrine-related items (sleep, fatigue, nervousness and nausea) are already included in the HR-QOL items. The result of these four items in addition to the 19 items of the endocrine subscale can also be scored as an additional and more extended endocrine subscale (ES23, 23 items).^20^ The different endocrine subscale items can be found in Supplementary Material S1, available at https://doi.org/10.1016/j.esmoop.2023.100786. Higher scores of the FACT-ES equate with good quality of life and/or experiencing few side-effects, while lower scores indicate poorer quality of life and/or experiencing many/severe side-effects. As an additional insight into the effect of dose reduction, patients were asked to score the beneficial effect of their dose reduction on a 10-point Likert scale (range 1-10, 1: no improvement, 10: excellent improvement) 3 months after the tamoxifen dose was reduced.

### Statistical analysis

The primary endpoint of this study was the individual difference in total ES19 score as part of the FACT-ES questionnaire in patients whose tamoxifen dose was reduced before and after 3 months of tamoxifen dose reduction. Change scores of >0.5 of the baseline standard deviation (SD) are considered clinically relevant changes and seen as more than a moderate effect size.^21^ Before the start of the study, it was estimated that the SD would be 7-8 points and therefore a change score of minimally 4 points, also used in an earlier endocrine subscale validation study, was expected to be clinically relevant.^20^ We hypothesized that the ES19 score would improve with at least 4 points in >50% of dose-reduced patients. To test this hypothesis against a null hypothesis of 20% with a one-sided α of 0.05 and a power of 80%, the tamoxifen dose had to be reduced in at least 13 patients.

The primary endpoint was analysed by means of the binomial probability test. The percentage of patients with a reduction in experienced side-effects of >0.5 of baseline SD will be given together with the binomial exact 90% confidence interval (CI).

As secondary endpoints, the individual differences in ES23 and HR-QOL measured with the FACT-ES before and after dose reduction were determined and compared with >0.5 of baseline SD that was found in this study. Also, the within-group difference before and 3 months after dose reduction was determined with a paired sample *t*-test or a Wilcoxon signed rank test when a sample was not normally distributed. To check for a potential effect of time on tamoxifen-related side-effects, analyses were repeated in the group of patients with side-effects who remained on the regular tamoxifen 20 mg dose between 3 and 6 months of treatment. Differences in specific side-effects (separate items of the extensive endocrine subscale) were analysed descriptively. In an exploratory way, we searched for a correlation between change in FACT-ES scores before and after dose reduction and endoxifen levels at 3 months of tamoxifen 10 mg use.

## Results

### Patient selection

In total, 151 patients were included in this secondary aim of the TOTAM trial. Two patients were excluded due to not yet reaching a steady-state endoxifen level or accidentally using a lower tamoxifen dose. Of the 149 assessable patients, 125 (84%) patients experienced any tamoxifen-related side-effect and scored ≤72 points on the endocrine subscale. Of these 125 patients, 37 patients (30%) had an endoxifen level ≥32 nM and 18 of these patients experienced their side-effects as bothersome and thus were eligible for tamoxifen dose reduction. Later on in the study, two additional patients who at 3 months of therapy had endoxifen levels just below 32 nM but at the time of dose reduction had endoxifen levels ≥32 nM and bothersome side-effects underwent a reduction in tamoxifen dose. Eventually, in 20 out of 149 (13%) patients in this study, tamoxifen dose was reduced and of these patients, 17 were assessable for the primary endpoint. This number is slightly higher than the minimum number of patients required for this study (*n* = 13). This difference occurred because eligible patients were offered dose reduction until at least 13 assessable patients used tamoxifen 10 mg for at least 3 months. Interestingly, also four patients who at baseline refused a dose reduction asked for dose reduction themselves after a longer duration of tamoxifen treatment. A flowchart visualizing the selection process can be found in Supplementary Material S2, available at https://doi.org/10.1016/j.esmoop.2023.100786. All patients with side-effects, an endocrine subscale score ≤72 and endoxifen levels ≥16 nM who remained on tamoxifen 20 mg from baseline until 6 months of tamoxifen (*n* = 60) were used as a control group, which also can be found in Supplementary Material S2, available at https://doi.org/10.1016/j.esmoop.2023.100786.

### Patient characteristics

The baseline characteristics of the 17 assessable patients after dose reduction can be found in Table 1. For comparison, the baseline characteristics of the 60 patients with side-effects who remained on tamoxifen 20 mg from baseline until 6 months of tamoxifen use are also shown. Patients in both the 20-mg and 10-mg groups had a median age of 59 years. The incidence of (neo)adjuvant chemotherapy treatment was similar between both groups. The use of ovarian function suppression (OFS) was very low but slightly higher in the dose-reduction group. After a median of 113 days of tamoxifen use, patients underwent a reduction in their tamoxifen dose.

**Table 1:** Patient characteristics

	Dose-reduction cohort (*n* = 17)	Patients with side-effects who remained on tamoxifen 20 mg (*n* = 60)
Age (years), median (IQR)	59 (49-64)	59 (49-66)
BMI (kg/m^2^), median (IQR)	25.8 (21.8-30)	26.2 (23.2-30.5)
Tumour stage, *n* (%)		
T1	10 (59)	27 (45)
T2	7 (41)	23 (38.3)
T3	—	8 (13.3)
T4	—	1 (1.7)
Tx	—	1 (1.7)
Nodal stage, *n* (%)		
N0	11 (65)	33 (55)
N1	5 (29)	19 (31.7)
N2	1 (6)	6 (10)
N3	—	2 (3.3)
Histologic classification, *n* (%)		
Ductal (NST)	15 (88)	44 (73.3)
Lobular	2 (12)	13 (21.7)
Other	—	3 (5)
Histologic grade (Bloom Richardson), *n* (%)		
BR I	3 (18)	5 (8.3)
BR II	11 (65)	44 (73.3)
BR III	3 (18)	11 (18.3)
Progesterone receptor, *n* (%)		
Positive	13 (80)	52 (86.7)
Negative	4 (20)	8 (13.3)
Her2Neu receptor, *n* (%)		
Positive	1 (6)	3 (5)
Negative	16 (94)	57 (95)
Local treatment, *n* (%)		
Lumpectomy alone	1 (6)	1 (1.7)
Lumpectomy + RTx	14 (82)	36 (60)
Mastectomy	1 (6)	11 (18.2)
Mastectomy + RTx	1 (6)	12 (20)
(Neo)adjuvant chemotherapy, *n* (%)		
Yes	8 (47)	30 (50)
No	9 (53)	30 (50)
CYP2D6 phenotype, *n* (%)		
PM	—	—
IM	5 (29)	18 (30)
NM	10 (59)	42 (70)
UM	2 (12)	—
Use of ovarian function suppression (OFS), *n* (%)		
Yes	2 (11.8)	5 (8.3)
No	15 (88.2)	55 (91.7)
Days of tamoxifen before dose reduction, median (IQR)	113 (101-154)	—

BMI, body mass index; IQR, interquartile range.

### FACT-ES scores before and after 3 months of dose reduction

FACT-ES scores were assessed after a median of 91 days [interquartile range (IQR) 85-95 days] after dose reduction. The baseline SD of the ES19 was higher than expected and therefore an improvement of >0.5 SD (i.e. clinically relevant) was equal to 6 points. Out of the 17 assessable patients in whom the tamoxifen dose was reduced, 7 patients (41%, 90% CI 21% to 65%, *P* = 0.038) had an improvement in ES19 score of at least 6 points. The ES23 score had to improve with ≥7 points to be clinically relevant, which was achieved in 5 out of 17 patients (29%, 90% CI 12% to 52%). The HR-QOL improved with 6 points or more (>0.5 SD) in 11 out of 17 patients (65%, 90% CI 42% to 83%). Change scores of all individual patients in ES19, ES23 and HR-QOL can be found in Table 2. There was a significant and clinically relevant within-group improvement in HR-QOL after dose reduction compared with scores before dose reduction (Table 3). There was also a clinically relevant improvement in ES19 and ES23 and this improvement had a trend towards statistical significance (*P* = 0.053).

**Table 2:** Individual change scores in FACT-ES

Subject	Change in scores after 3 months of dose reduction		
	HR-QOL	Endocrine subscale 19	Endocrine subscale 23
1	18	23	26
2	2	−1	−3
3	11	3	2
4	−6	2	5
5	19	9	6
6	6	1	1
7	16	21	24
8	−5	−3	−2
9	45	14	23
10	18	10	11
11	−4	1	0
12	5	7	6
13	−21	−12	−13
14	8	−2	1
15	9	−9	−7
16	12	30	36
17	6	3	4
**0.5 SD**	**5.65**	**5.35**	**6.05**

Change in scores of FACT-ES questionnaire after 3 months of tamoxifen dose reduction compared to FACT-ES scores at steady-state tamoxifen 20 mg. Negative change scores stand for worsening of symptoms, while positive change scores stand for improvement of symptoms. Change scores of >0.5 baseline SD are considered clinically relevant.

FACT-ES, Functional Assessment of Cancer Therapy—Endocrine Symptoms; HR-QOL, health-related quality of life; SD, standard deviation.

**Table 3:** Within-group differences after dose reduction or between 3 and 6 months of tamoxifen treatment

Before and after dose reduction (*n* = 17)	Mean before dose reduction	Mean 3 months after dose reduction	Difference before and after dose reduction (95% CI)	*P* value (two-sided)
Health-related QOL	74.3	82.5	8.2 (0.9-15.4)	0.03
Endocrine subscale 19	49.3	55.0	5.7 (−0.5-11.5)	0.053
Endocrine subscale 23	58.9	66.0	NA^a^	0.053

3 and 6 months of tamoxifen 20 mg use (*n* = 60)	Mean 3 months	Mean 6 months	Δ 6 months versus 3 months (95% CI)	*P* value (two-sided)
Health-related QOL	82.6	81.9	−0.6 (−2.8-1.5)	0.55
Endocrine subscale 19	56.2	56.7	0.5 (−1.4-2.4)	0.61
Endocrine subscale 23	68.0	68.5	0.5 (−1.9-2.8)	0.71

CI, confidence interval; QOL, quality of life.

a Wilcoxon signed rank test

### FACT-ES scores at 3 and 6 months of tamoxifen 20 mg use

To check for a potential effect of time on side-effects, the FACT-ES scores after 3 months of tamoxifen were compared with the FACT-ES scores after 6 months of tamoxifen in patients who had side-effects and remained on the standard dose of tamoxifen 20 mg. There was no statistically or clinically relevant within-group difference in FACT-ES scores at 6 months of tamoxifen compared to 3 months of tamoxifen. CIs and *P* values can be found in Table 3. Also, when making a sub-selection of patients with endoxifen levels ≥32 nM who remained on tamoxifen 20 mg (*n* = 16), no difference in FACT-ES scores between 3 and 6 months of tamoxifen was found (data not shown). In 11 out of 60 (18%, 90% CI 11% to 29%) patients, the HR-QOL improved with at least 6 points. In 13 out of 60 patients (22%, 90% CI 13% to 32%), the ES19 improved with at least 5 points and in 14 out of 60 patients (23%, 90% CI 15% to 34%) the ES23 improved with 6 points or more (i.e. clinically relevant improvements).

### Side-effects before and after dose reduction

Adverse events before and after dose reduction are shown in Table 4. The majority of side-effects decreased after tamoxifen dose reduction. However, the incidence of muscle cramp and weight gain remained the same and fatigue, anxiety, increased appetite and vaginal discharge all occurred in one additional patient despite dose reduction. Two patients had a CTCAE 2 and a CTCAE 3 graded thromboembolic event after tamoxifen was started but before tamoxifen dose was reduced and this was also partly the reason to decrease their tamoxifen dose.

**Table 4:** CTCAEv5 toxicity before and after dose reduction

Side-effect	Before dose reduction at steady-state tamoxifen 20 mg (*n* = 17) *n* (%)		3 months after dose reduction to tamoxifen 10 mg (*n* = 17) *n* (%)
	CTCAE 1	CTCAE 2	CTCAE 1
Hot flashes	13 (76)	2 (12)	12 (71)
Insomnia	10 (59)	1 (6)	7 (41)
Mood alterations	10 (59)	0	4 (24)
Arthralgia	7 (41)	3 (18)	8 (47)
Muscle cramp	4 (24)	0	4 (24)
Nausea	2 (12)	1 (6)	1 (6)
Headache	3 (18)	0	1 (6)
Vaginal dryness	3 (18)	0	1 (6)
Fatigue	2 (12)	0	3 (18)
Dizziness	2 (12)	0	1 (6)
Weight gain	2 (12)	0	2 (12)
Chills	1 (6)	0	0
Bloated feeling	1 (6)	0	0
Obstipation	1 (6)	0	1 (6)
Alopecia	1 (6)	0	1 (6)
Dry mouth	1 (6)	0	0
Anorexia	1 (6)	0	0
Decreased libido	1 (6)	0	0
Amnesia	1 (6)	0	0
Increased appetite	0	0	1 (6)
Anxiety	0	0	1 (6)
Vaginal discharge	0	0	1 (6)

CTCAEv5, Common Terminology Criteria for Adverse Events version 5.

Patients were asked to score the improvement of side-effects 3 months after dose reduction on a 10-point Likert scale. Twelve patients (71%) graded their improvement as sufficient whereof seven patients scored a 6-7 and five patients scored an 8 or higher.

Finally, the effect of dose reduction on separate items of the ES23 was analysed descriptively. In contrast to the CTCAEv5 grading, the improvement was most frequently seen in the lack of energy item (*n* = 10; 59%). Other frequently improved items were hot flashes and cold sweats (*n* = 9; 53%) and night sweats, insomnia, bloated feeling, mood swings and lightheaded feeling (*n* = 8; 47%).

### Effect of dose reduction on tamoxifen and endoxifen plasma levels

**Figure 1:**
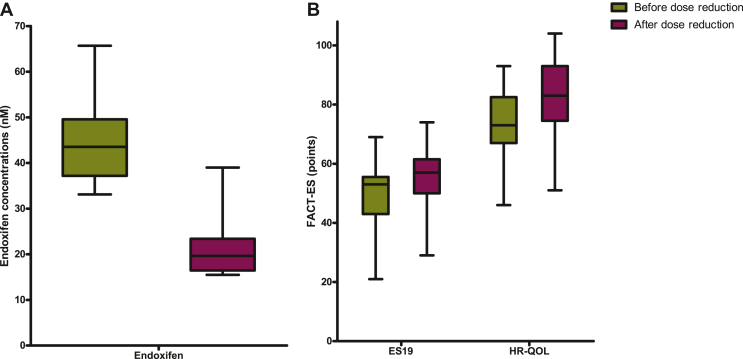
**Effect of dose reduction on endoxifen concentrations, endocrine symptoms and quality of life.** (A) Median, IQR, minimum and maximum endoxifen concentrations before and after 3 months of dose reduction (*n* = 18). (B) Median, IQR, minimum and maximum ES19 scores and HR-QOL scores before and after 3 months of dose reduction (*n* = 17). ES19, endocrine subscale of in total 19 items; FACT-ES, Functional Assessment of Cancer Therapy—Endocrine Symptoms; HR-QOL, health-related quality of life; IQR, interquartile range.

The median tamoxifen level of the patients at 20 mg daily who underwent a reduction in tamoxifen dose was 397 nM (IQR 343.7-478.8 nM) and the median endoxifen level was 43.2 nM (IQR 37.1-49.0 nM) before dose reduction. One patient quit tamoxifen treatment before 3 months of tamoxifen 10 mg were completed and was therefore not assessable for pharmacokinetic analysis. Endoxifen levels of 15 out of 19 patients (79%) remained above the supposed threshold of 16 nM after 3 months of tamoxifen 10 mg. One patient had already an endoxifen level below 16 nM (12.8 nM) after 26 days of dose reduction. Three patients had endoxifen levels ranging from 15.5 to 15.9 nM after 3 months of tamoxifen 10 mg use. All four patients whose endoxifen levels fell below 16 nM after the 50% dose reduction had baseline endoxifen levels just above the accepted lower boundary of 32 nM (endoxifen levels ranging from 35 to 37.7 nM). The median tamoxifen level in the 18 patients measured 3 months after tamoxifen 10 mg was 216.5 nM (IQR 163.3-285.3 nM) and the median endoxifen level was 19.7 nM (IQR 16.5-23.4 nM). A visual illustration of the effect of dose reduction on endoxifen levels and FACT-ES scores is shown in Figure 1.

### Correlation between endoxifen levels and the effect of dose reduction on side-effects

There was a significant moderately negative correlation between change in ES19 scores after dose reduction and endoxifen levels after 3 months of tamoxifen 10 mg (*r* = −0.68, *P* = 0.003, *n* = 17) meaning that more improvement after dose reduction was seen in patients who attained lower endoxifen levels after dose reduction. Change in HR-QOL even showed a significant highly negative correlation with endoxifen levels after 3 months of tamoxifen 10 mg (*r* = −0.72, *P* = 0.001, *n* = 17).

## Discussion

To the best of our knowledge, this is the first study describing the effects of adjuvant endoxifen-guided tamoxifen dose reductions on a broad selection of therapy-related side-effects based on a validated questionnaire. We demonstrated that reducing the tamoxifen dose improves endocrine symptoms in almost half of the patients and strongly increases HR-QOL (even in two-thirds of patients). This improvement does not occur over time in patients with side-effects who remained on tamoxifen 20 mg. Of the patients who underwent a dose reduction, 79% retained endoxifen levels well above the conservative threshold of 16 nM, while 16% had endoxifen levels just below 16 nM (15.5-15.9 nM). Only one patient dropped ∼20% below the most conservative threshold of 16 nM. Thus, endoxifen-guided dose reduction of tamoxifen, with the aim of alleviating symptoms, led to significant and clinically relevant conditions in a significant proportion of women.

In this study, we chose to only carry out dose reductions in patients with endoxifen levels ≥32 nM. Since tamoxifen has linear pharmacokinetics, we tried to retain almost all patients above the 16 nM threshold. Considering the (low) intra-patient endoxifen variability of ∼10%-20%, it is not surprising that some patients’ endoxifen plasma levels still dropped slightly below 16 nM after halving the tamoxifen dose.^18^^,^^22^ Indeed, all four patients whose endoxifen levels fell below 16 nM had baseline endoxifen levels only slightly above the accepted lower boundary of 32 nM. As mentioned before, the threshold of 16 nM is a conservative one and other threshold values have been mentioned before in studies as well.^12^, ^13^, ^14^ However, since this is a curative setting, it is extremely important to maintain effective endoxifen levels. Therefore, if patients drop below 16 nM after dose reduction, a slight increase of the mean dose of tamoxifen to, for example, 15 mg daily should be considered.

Unfortunately, this study does not offer a solution for patients with endoxifen levels <32 nM in case they suffer from tamoxifen-related side-effects. But, if we take the intra-patient variability into account, dose reduction in case of severe side-effects could eventually also be tried when patients have endoxifen levels within 26-32 nM (20% range) and endoxifen levels could then possibly still remain above or around 16 nM. Another option might be to try dose reduction from 20 mg to 15 mg in patients with severe side-effects and endoxifen levels ranging between 21 and 32 nM. Consequently, 2-3 months after dose reduction, endoxifen levels should be measured again and the effect of dose reduction on side-effects should be assessed. If endoxifen levels with tamoxifen 20 mg are already far below 32 nM, endoxifen levels after dose reduction drop far below the conservative level of 16 nM or if patients do not benefit from tamoxifen dose reduction, another strategy, like switching to aromatase inhibitors, should be considered.

The improvement in endocrine symptoms seen in this study was, although clinically relevant and significant, less than hypothesized in advance. Any improvement in ES19 was seen in 12 out of 17 (71%) dose-reduced patients while a clinically relevant improvement (≥6 points) was seen in 41% of patients. A possible explanation for this lower than hypothesized improvement is the use of OFS next to tamoxifen in part of premenopausal women. OFS has quite similar side-effects as tamoxifen and when tamoxifen dose is reduced this clearly does not affect side-effects due to OFS. Two patients used OFS and both did not achieve clinically relevant improvements in ES19 and ES23 scores after dose reduction. Secondly, patients could have other side-effect-inducing events during tamoxifen dose reduction. For instance, one patient used amoxicillin/clavulanic acid for 2 weeks because of an undefined infection during the third month of tamoxifen dose reduction and showed a decrease in ES19, ES23 and HR-QOL scores.

Most remarkably, we found a correlation between endoxifen levels after 3 months of tamoxifen 10 mg and change in HR-QOL and ES19 scores with more improvement in FACT-ES scores when endoxifen levels after 3 months of 10 mg were lower and less improvement in FACT-ES scores when endoxifen levels still remained relatively high. Possibly, patients whose endoxifen level remains relatively high after dose reduction could benefit from even further tamoxifen dose de-escalation.

In some patients, the endocrine side-effects did not improve in a clinically relevant way while HR-QOL did improve. This is most likely the result of an improvement in HR-QOL resulting from causes other than the reduction of endocrine therapy-related symptoms, such as recovery from breast surgery and possibly chemotherapy. However, this improvement in HR-QOL was not seen in the group of patients who remained on standard dose of tamoxifen. An alternative explanation would be that a very small improvement of side-effects can significantly improve quality of life during long adjuvant treatment of tamoxifen.

In an earlier study from our group, the feasibility of therapeutic drug monitoring of tamoxifen was shown.^18^ Here, tamoxifen dose escalation in patients with endoxifen levels <16 nM nearly halved the percentage of patients with endoxifen levels below the threshold. Our current study complements nicely to this preceding research, offering a solution for patients on the other side of the spectrum (i.e. patients having high levels of endoxifen with sometimes severe side-effects). Our study highlights once again how important it is to know the ‘true’ threshold of endoxifen to implement this in therapeutic drug monitoring of tamoxifen in clinical practice. However, in order to confirm an endoxifen exposure–response relationship prospectively, large trials of over 3000 patients would be needed, making this kind of proof for an endoxifen threshold infeasible.^23^^,^^24^ Until further evidence is attained, for example, from neoadjuvant window-of-opportunity trials, the conservative endoxifen threshold of 16 nM could be used pragmatically for tamoxifen dose escalation in patients with endoxifen levels <16 nM and for dose de-escalating (dose reduction) in patients with severe side-effects.

Of course, our study has some limitations. Firstly, a placebo effect of lowering the tamoxifen dose cannot be ruled out as the dose adjustment was not blinded. Secondly, this was not a randomized study. Dose reduction was offered to all patients with bothersome side-effects and endoxifen levels ≥32 nM and patients were free in making a choice for dose reduction or remaining on 20-mg dose. This led to different time points of dose reduction and it could be possible that patients who had more belief in improvement from this intervention were more likely to choose for dose reduction, introducing potential bias. However, the different time points of dose reduction also account for more robustness of the study and make it more convertible to daily practice. In this study, a relatively limited number of dose-reduced cases are discussed. This might lead to less generalizability, although of course the linear pharmacokinetics of tamoxifen is the same for every patient. Another limitation is the duration of the study. Because we only evaluated side-effects after 3 months, this study does not provide information about durability of the improvement. Probably, side-effects could improve further over time making the impact of tamoxifen dose reduction even bigger. Lastly, we chose to only investigate dose reductions from 20 mg to 10 mg. Therefore, we lack interesting information about side-effects after dose adjustments to tamoxifen 15 mg.

### Conclusions

In conclusion, we demonstrated that endoxifen-guided dose reduction in case of bothersome tamoxifen-related side-effects can improve endocrine symptoms in almost half of the patients and strongly increase HR-QOL in two-thirds of these patients while keeping endoxifen levels mainly above or around the threshold. Therefore, it could be an effective strategy for patients who would otherwise quit their endocrine therapy or who are highly suffering from tamoxifen-related side-effects.
