# Supplementary material for: The impact of endoxifen-guided tamoxifen dose reductions on endocrine side-effects in patients with primary breast cancer
Source: ESMO Open. 2023 Feb 6;8(1):100786. doi: 10.1016/j.esmoop.2023.100786 (PMC10024121; doi:10.1016/j.esmoop.2023.100786)
Supplement: Supplementary material 1 [file mmc1.docx]

**Supplementary information**

**FACT-ES items ES19 and ES23**

**Legend.** *The FACT-ES has a 5 point liker-type response scale and measures four domains of health-related quality of life: physical, social, emotional and functional well-being (i.e. FACT-General). The additional endocrine-subscale specifically measures hormone therapy related side effects with 19 (ES19, i.e. the standard version) or 23 (ES23, i.e. the extended version) items. Some items were negatively framed and were therefore reversed for analysis.*

| Endocrine subscale items FACT-ES | | | | | |
| --- | --- | --- | --- | --- | --- |
| ES19 items | Not at all | A little bit | Some-what | Quite a bit | Very much |
| I have hot flashes | 0 | 1 | 2 | 3 | 4 |
| I have cold sweats | 0 | 1 | 2 | 3 | 4 |
| I have night sweats | 0 | 1 | 2 | 3 | 4 |
| I have vaginal discharge | 0 | 1 | 2 | 3 | 4 |
| I have vaginal itching/irritation | 0 | 1 | 2 | 3 | 4 |
| I have vaginal bleeding or spotting | 0 | 1 | 2 | 3 | 4 |
| I have vaginal dryness | 0 | 1 | 2 | 3 | 4 |
| I have pain or discomfort with intercourse | 0 | 1 | 2 | 3 | 4 |
| I have lost interest in sex | 0 | 1 | 2 | 3 | 4 |
| I have gained weight | 0 | 1 | 2 | 3 | 4 |
| I feel lightheaded/dizzy | 0 | 1 | 2 | 3 | 4 |
| I have been vomiting | 0 | 1 | 2 | 3 | 4 |
| I have diarrhea | 0 | 1 | 2 | 3 | 4 |
| I get headaches | 0 | 1 | 2 | 3 | 4 |
| I feel bloated | 0 | 1 | 2 | 3 | 4 |
| I have breast sensitivity/tenderness | 0 | 1 | 2 | 3 | 4 |
| I have mood swings | 0 | 1 | 2 | 3 | 4 |
| I am irritable | 0 | 1 | 2 | 3 | 4 |
| I have pain in my joints | 0 | 1 | 2 | 3 | 4 |
| ES23 items (ES19 + four items below) | 0 | 1 | 2 | 3 | 4 |
| I have a lack of energy | 0 | 1 | 2 | 3 | 4 |
| I have nausea | 0 | 1 | 2 | 3 | 4 |
| I feel nervous | 0 | 1 | 2 | 3 | 4 |
| I am sleeping well | 0 | 1 | 2 | 3 | 4 |
