# Supplementary material for: The impact of endoxifen-guided tamoxifen dose reductions on endocrine side-effects in patients with primary breast cancer
Source: ESMO Open. 2023 Feb 6;8(1):100786. doi: 10.1016/j.esmoop.2023.100786 (PMC10024121; doi:10.1016/j.esmoop.2023.100786)
Supplement: Supplementary material 2 [file mmc2.pptx]

## Slide 1
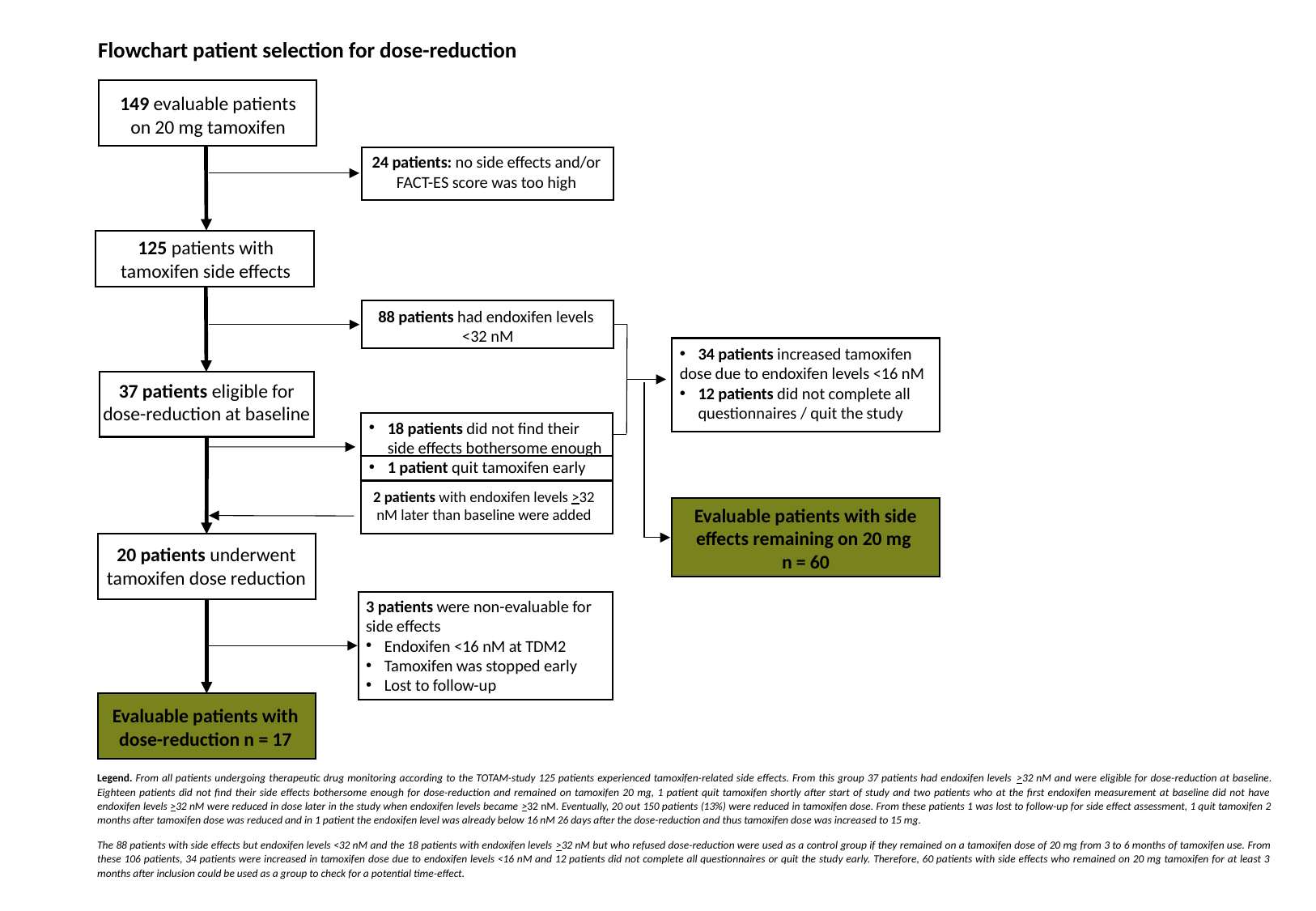

Flowchart patient selection for dose-reduction
149 evaluable patients on 20 mg tamoxifen
24 patients: no side effects and/or FACT-ES score was too high
125 patients with tamoxifen side effects
88 patients had endoxifen levels
<32 nM
34 patients increased tamoxifen
dose due to endoxifen levels <16 nM
12 patients did not complete all questionnaires / quit the study
37 patients eligible for dose-reduction at baseline
18 patients did not find their side effects bothersome enough
1 patient quit tamoxifen early
2 patients with endoxifen levels >32 nM later than baseline were added
Evaluable patients with side effects remaining on 20 mg
n = 60
20 patients underwent tamoxifen dose reduction
3 patients were non-evaluable for side effects
Endoxifen <16 nM at TDM2
Tamoxifen was stopped early
Lost to follow-up
Evaluable patients with dose-reduction n = 17
Legend. From all patients undergoing therapeutic drug monitoring according to the TOTAM-study 125 patients experienced tamoxifen-related side effects. From this group 37 patients had endoxifen levels >32 nM and were eligible for dose-reduction at baseline. Eighteen patients did not find their side effects bothersome enough for dose-reduction and remained on tamoxifen 20 mg, 1 patient quit tamoxifen shortly after start of study and two patients who at the first endoxifen measurement at baseline did not have endoxifen levels >32 nM were reduced in dose later in the study when endoxifen levels became >32 nM. Eventually, 20 out 150 patients (13%) were reduced in tamoxifen dose. From these patients 1 was lost to follow-up for side effect assessment, 1 quit tamoxifen 2 months after tamoxifen dose was reduced and in 1 patient the endoxifen level was already below 16 nM 26 days after the dose-reduction and thus tamoxifen dose was increased to 15 mg.
The 88 patients with side effects but endoxifen levels <32 nM and the 18 patients with endoxifen levels >32 nM but who refused dose-reduction were used as a control group if they remained on a tamoxifen dose of 20 mg from 3 to 6 months of tamoxifen use. From these 106 patients, 34 patients were increased in tamoxifen dose due to endoxifen levels <16 nM and 12 patients did not complete all questionnaires or quit the study early. Therefore, 60 patients with side effects who remained on 20 mg tamoxifen for at least 3 months after inclusion could be used as a group to check for a potential time-effect.
